# Implications of COVID-19 pandemic on internet addiction among Croatian university students

**DOI:** 10.1192/j.eurpsy.2023.827

**Published:** 2023-07-19

**Authors:** M. Miskulin, N. Pavlovic, I. Miskulin, J. Kovacevic

**Affiliations:** Department of Public Health, Faculty of Medicine Osijek, Osijek, Croatia

## Abstract

**Introduction:**

During the COVID-19 pandemic the internet has become an important medium for learning and communication for university students. Increased time spent online during the pandemic is a significant risk factor for the development of internet addiction (IA) in this population.

**Objectives:**

This study aimed to investigate the influence of pandemics on IA among Croatian university students and to evaluate the characteristics of IA during the pandemic in comparison to pre-pandemic time.

**Methods:**

Two cross-sectional studies, one in April 2016 and another in April 2022 were conducted. As part of these studies, a validated, anonymous questionnaire that contained questions regarding demographic data, as well as Young’s Internet Addiction Test, was self-administered to a cross-faculty representative student sample of the University of Osijek in Eastern Croatia.

**Results:**

The study sample included 1602 university students (810 in the year 2016 and 792 in the year 2022), the median age was 21 years (interquartile range 20-22), 34.5% males, and 65.5% females. There was no statistically significant difference in the median age of the two students’ samples (p=0.234). The main reasons for internet usage were learning and faculty assignments (25.0%), social networking and entertainment (71.0%), and online gaming (4.0%), and there was no statistically significant difference between observed students’ samples (p=0.075). The overall prevalence of IA in 2016 was 41.4% and 39.8% in 2022 but this difference was not statistically significant (p=0.542). The proportions of students with mild, moderate, and severe IA in 2016 were 32.8%, 8.4%, and 0.1% respectively, while in 2022 they were 27.4%, 11.9%, and 0.5%, and observed differences were statistically significant (p=0.011).

**Conclusions:**

The COVID-19 pandemic has changed the pattern of IA among Croatian university students where proportions of students with moderate and severe IA were significantly higher in comparison to pre-pandemic time. To successfully manage this important public health challenge during the pandemic and post-pandemic time specific preventive measures intended for this population should be developed.

**Disclosure of Interest:**

None Declared

